# Telework during COVID-19 outbreak: Impact on mental health among Italian workers

**DOI:** 10.1192/j.eurpsy.2021.1799

**Published:** 2021-08-13

**Authors:** V. Bertino, V. Nisticò, A. D’Agostino, O. Gambini, B. Demartini

**Affiliations:** 1 Unità Di Psichiatria Ii, ASST Santi Paolo e Carlo, Presidio San Paolo, Milano, Italy; 2 Dipartimento Di Scienze Della Salute, Università degli Studi di Milano, Milano, Italy; 3 “aldo Ravelli” Research Center For Neurotechnology And Experimental Brain Therapeutics, Università degli Studi di Milano, Milano, Italy

**Keywords:** stress, Teleworking, Anxiety, Depression

## Abstract

**Introduction:**

Since February 2020, the outbreak of COVID-19 has spread to several countries worldwide, including Italy, leading to an uptake of telework.

**Objectives:**

We aim to evaluate the psychopathological impact of teleworking during the COVID-19 pandemic in Italy, identifying mental health determinants among home-based workers.

**Methods:**

804 participants completed an online survey, including the psychometric scales “Depression, Anxiety and Stress Scale – 21 items” (DASS-21) and the “Insomnia Severity Index” (ISI). Teleworkers were also asked to provide information about their current work routine, home environment and clinical history.

**Results:**

At the DASS-21, 30% of the participants presented pathological levels of depression, 20.8% of anxiety and 30.7% of stress. At the ISI, 5% appeared to suffer from insomnia. Respondents with psychological and physical frailties, greater social isolation or inadequate working spaces manifested higher levels of psychiatric symptoms. Moreover, we also find a correlation of these symptoms with occupations in education. Telework was broadly appreciated and 87% of respondents expressed a willingness to maintain access to this arrangement.

**Conclusions:**

Our results document that about a third of our sample manifested psychopathological symptoms while teleworking during the COVID-19 outbreak in Italy. However, telework itself does not seem to be directly associated with increased psychiatric symptoms, which were instead exacerbated by COVID-19-related stressful circumstances, as well as by constitutional and social determinants of health. Going forward, authorities should promote adequate measures in order to guarantee a healthy approach to teleworking.

**Disclosure:**

No significant relationships.

